# Gender Differences in the Associations Between Perceived Parenting Styles and Young Adults’ Cyber Dating Abuse

**DOI:** 10.3389/fpsyg.2022.818607

**Published:** 2022-03-24

**Authors:** F. Giorgia Paleari, Laura Celsi, Desirèe Galati, Monica Pivetti

**Affiliations:** Department of Human and Social Sciences, University of Bergamo, Bergamo, Italy

**Keywords:** parenting styles, cyber dating abuse, gender differences, young adults, gendered socialization

## Abstract

Existing literature indicates that parenting styles affect the development of cyber aggression in offspring differently, depending on the gender of children. The present study investigates whether mothers’ and fathers’ parenting styles show similar gender differences in their associations with a new form of dating violence, i.e., cyber dating abuse (CDA). The limited evidence on the issue focuses on the relation that each parenting style has with CDA perpetration, without considering CDA victimization and the joint effects of fathers’ and mothers’ parenting styles. The present study contributes to the research on gender differences in parenting by examining whether young adults’ perceptions of maternal and paternal parenting styles during childhood were independently and/or jointly related to their perpetrated and suffered CDA and whether these relations differed across young adults’ gender. In total, 351 young adults (50.7% men), age between 18 and 35 years and having a romantic relationship, completed online self-reports of the variables of interest that include a bidimensional measure of perpetrated/suffered CDA that assess aggression and control. Results showed that maternal authoritarian parenting was uniquely and positively associated to their children’s perpetration and victimization of cyber dating control, whereas maternal permissive parenting was uniquely and positively related to their children’s perpetration of cyber dating aggression and victimization of cyber dating control. For daughters, these associations were stronger when the father’s style was similar to the mother’s one or when a maternal authoritarian style combined with a paternal permissive style, thus indicating that the two parents’ parenting styles interact in relating to their daughters’ CDA.

## Introduction

The family of origin usually is the most important socialization agent in the early stages of individuals’ development. Several theoretical models, such as social learning theory (Bandura, 1977), coercion theory (Reid et al., 2002), attachment theory (Michiels et al., 2008), and the self-determination theory (Soenens et al., 2015), suggest that parents may affect children’s and adolescents’ behaviors and their peer relationships through their parenting choices, practices, and beliefs.

According to Baumrind (1971, 1991), the construct that best summarizes the main factors through which parents influence the socio-emotional and behavioral development of their children is parenting style, that is the pervasive emotional climate within which the child is raised (Darling and Steinberg, 1993). Baumrind’s (1971, 1991) categorical model distinguishes three different parenting styles–authoritative, authoritarian, and permissive–on the basis of four parental behavior dimensions: parental warmth, control, demand, and involvement. More specifically, authoritative style, characterized by high levels of control, demand, parental warmth, and involvement, is recognizable in affective and sensitive parents, who discipline their children through open communication and example, have high but reasonable demands, and are strict but fair. The authoritarian style, characterized by high levels of control and demand and low levels of warmth and involvement, is evident in strict and inflexible parents, who show high expectations toward their children, little sensitivity toward emotional needs of children, and punish them without explaining the meaning of the rules imposed. Finally, the permissive style, characterized by high levels of parental warmth and involvement and low levels of control and demand, is identifiable in caring, affective, and sensitive parents, who exercise the role of friends rather than parents and thus display an excessive indulgence and poor ability in the exercise of normative functions.

The wide empirical literature inspired by this model generally attests that the authoritarian and, to a lesser degree, the permissive parenting style contribute to the development of behavioral problems, such as the perpetration of bullying and dating violence in offspring (Luk et al., 2016; Olivari et al., 2017; Pinquart, 2017; Cuccì et al., 2019; Ruiz-Hernández et al., 2019; Moreno Méndez et al., 2020). Conversely, the authoritative parenting has a protective effect against externalizing problems and both perpetration and victimization of relationship abuse, even in the presence of parental inconsistency (Luk et al., 2016; Mumford et al., 2016; Pinquart, 2017; Ruiz-Hernández et al., 2019). These effects resulted not moderated by child and parent gender (Pinquart, 2017). The cultural invariance of the above findings was, however, questioned by recent research in Latin American and Mediterranean European countries, where permissive parenting was found to have more positive outcomes than expected (Martínez et al., 2019; Suárez-Relinque et al., 2019).

The digital revolution has caused such substantial changes within relational dynamics, especially among current adolescents and young adults belonging to the Y and the Z generations (Buckingham and Willett, 2006; Junco and Mastrodicasa, 2007), that scholars have been forced to rethink the construct of violence in a way that also includes the virtual world. Recent studies have indeed highlighted the rapid spread in cyber space of new forms of intentional acts harming individuals or groups, which has been given the name of cyber aggression (Zhao and Gao, 2012; Zhang et al., 2021). Results available to date on the role of parenting styles in predicting offspring cyber aggression are only partially consistent with those concerning violence in the real world. In fact, several studies show that the parental authoritarian style positively relates to children’s perpetration and victimization of cyber aggression; however, the relation between authoritarian parenting and perpetrated cyber aggression relation seems stronger for men than for women, suggesting that the authoritarian style fosters greater assimilation of traditional gender roles in which violence is less criticized in boys (Elsaesser et al., 2017; He et al., 2017; Martínez-Ferrer et al., 2019; Moreno-Ruiz et al., 2019; Zhang et al., 2021). In addition, results linking cyber aggression to the other two parenting styles seem more inconsistent: some reveal that parental indulgent and authoritative styles relate negatively with cyber violence, whereas some others indicate they are unrelated or positively related to it (e.g., Vale et al., 2018; Moreno-Ruiz et al., 2019; Zhang et al., 2021).

Among the various forms of cyber aggression, cyber dating abuse (CDA) refers to acts of control, aggression, and sexual coercion that are digitally perpetrated against the romantic partner through new media, such as social network sites, text messages, emails, or technology, such as geolocation app (Zweig et al., 2013, 2014; Borrajo et al., 2015; Reed et al., 2017). CDA appears to be widespread and dangerous for the mental health of both victims and perpetrators, resulting in externalizing and internalizing symptoms (Drauker and Martsolf, 2010; Bennet et al., 2011; Zweig et al., 2014; Sargent et al., 2016; Flach and Deslandes, 2017; Van Ouytsel et al., 2017).

Regarding CDA etiology, some evidence suggests that adverse childhood experiences lived in the family, such as experiencing abuse and witnessing intimate partner violence (IPV), are related to an increased likelihood of CDA perpetration and victimization, directly or through the internalization of early maladaptive relational schemas (Celsi et al., 2021; Smith-Darden et al., 2016; Ramos et al., 2017). However, not much attention has been devoted to other family of origin factors that may contribute to CDA. Particularly, only one study by Muñiz-Rivas et al. (2019) has recently examined which parenting style best predicts the risk of CDA perpetration. Their findings indicate that male and female adolescents with authoritarian mothers were the most prone to inflict cyber dating aggression and cyber dating control, respectively, whereas adolescents with indulgent mothers were the less prone. The authors explained the greater influence of mothers’ parenting styles as the consequence of their greater involvement in daily child-rearing, especially in domains related to affective relationships. Indeed, mothers are expected to be and remain the main caregiver despite a steady increase in women’s participation in work outside of the home (Raley et al., 2012). Muñiz-Rivas et al. (2019), however, omit to assess CDA victimization and cyber sexual coercion and do not examine the joint effects of fathers and mothers’ parenting styles, despite there is evidence that the combination of the two parents’ styles can explain more variance in children’s externalizing behaviors than the focus on only one parent’s style (Berkien et al., 2012).

Informed by the literature just reviewed, the present research aimed at investigating whether young adults’ perceptions of maternal and paternal parenting styles during childhood were independently and/or jointly related to their perpetrated and suffered CDA, with focused attention on gender differences.

As for the unique relations of parenting styles with CDA, we hypothesized that independently of witnessing IPV between parents, the more young adults reported their mother or father as having been authoritarian, the more they perpetrated and suffered CDA (H1); authoritarian parenting was more strongly related to young adults’ perpetrated CDA when mothers’, rather than fathers’, parenting was considered (H2) and in men, rather than in women (H3). We were unable to make well-founded predictions about the association of permissive and authoritative styles with CDA, because of previous studies conflicting results relating those styles to cyber aggression and to CDA. Similarly, no specific predictions were made about the joint relations of parenting styles with CDA due to the lack of evidence on the issue.

## Methods

### Participants and Procedure

Participants were 351 young adults, 49.3% were women and 50.7% were men, aging on average 24 years (*M* = 24.20; *SD* = 3.20; range: 18–35). Their most frequent education qualifications were high school diploma or equivalent (46.4%), bachelor degree (28.8%), and master degree (21.1%).

All of them were engaged in a romantic relationship, mainly a heterosexual one (96.6%), averaging 3.62 years (*SD* = 2.99; range: 1 month–24 years). Most participants (79.2%) were not cohabiting with their romantic partners. All subjects had grown up with their parents.

On average, participants referred to use smartphones very often (*M* = 6.00; *SD* = 1.07) and social networks often (*M* = 5.24; *SD* = 1.36; possible range of response for both variables: from 1 = never to 7 = always).

Men and women did not differ with respect to any of the above socio-demographics except for social networks use, which was more frequent for women (*M* = 5.47) than for men [*M* = 5.02; *t*(349) = 3.147, *p* = 0.002].

Subjects were contacted through the publication of a post on instant messaging platforms, which presented the study as an anonymous survey on family and couple relationships and specified the inclusion criteria (identifying oneself as male or female, aging between 18 and 35 years, and having a romantic relationship lasting for at least 1 month). The message also contained a link to the online survey and asked participants to disseminate it to acquaintances. Informed consent was obtained from participants. The study complied with the Ethics Code of the Italian Psychology Association (Associazione Italiana di Psicologia [AIP], 2015) and was conducted in accordance with the (World Medical Association, 2013)-Declaration of Helsinki (1964/2013).

### Measures

#### Parenting Styles

Young adults’ perceptions of their parents’ parenting practices during childhood were measured through the 40-item Italian version of the Parenting Styles and Dimensions Questionnaire (PSDQ; Tagliabue et al., 2014). The participant responded to two versions of the scale, one for the mother’s parenting style and one for the father’s. The scale assesses the three parenting styles suggested by Baumrind (1971, 1991): authoritative (23 items, e.g., “My mother/father encouraged me to talk about my troubles”; α = 0.98 for both mothers and fathers) (see Supplementary Material for internal consistencies for men and women, separately), authoritarian (13 items, e.g., “My mother/father guided me by punishment more than by reason”; α = 0.92 and 0.94 for mothers and fathers, respectively), and permissive (4 items, e.g., “My mother/father stated punishments to me and did not actually did them”). Since the permissive subscale had shown low reliability in previous studies (e.g., Tagliabue et al., 2014), we increased it by adding to the subscale 10 more items from the original version of the PSDQ (Robinson et al., 2001) (α = 0.80 and 0.77 for mothers and fathers, respectively).

#### Perpetrated and Suffered Cyber Dating Abuse

Perpetrated and suffered CDA within the current romantic relationship was measured through a scale previously validated in Italy by Celsi et al. (2021). The scale consists of 40 items (20 for perpetration and 20 for victimization) assessing two dimensions of CDA: monitoring and control (11 items, e.g., “I/my partner checked my/my partner’s location and online activities”; α = 0.86 and 0.89 for perpetration and victimization, respectively) and psychological or sexual pressure and aggression (9 items, e.g., “I/my partner sent a threatening message to my partner/me”; α = 0.84 and 0.78 for perpetration and victimization, respectively).

#### Intimate Partner Violence Perpetrated by Parents

Physical and psychological IPV perpetrated by parents and witnessed by respondents during their childhood was assessed through a 6-item measure by Celsi et al. (2021). Three items measured violence perpetrated by the mother against the father and three items assessed violence perpetrated by the father against the mother (e.g., “I saw/heard my mother/father being insulted, denigrated, humiliated, or verbally assaulted by my father/mother”; α = 0.71 and 0.84 for violence perpetrated by mothers and fathers, respectively).

Participants responded to the items of the three measures using a 7-point Likert scale ranging from 1 (never) to 7 (always).

### Data Analysis

Hypotheses were verified using multiple regression analyses in SPSS, combined with Hayes’ (2013) PROCESS macros for Model 1, testing simple moderations (or 2-wave interactions), and Model 3, testing moderated moderations (or 3-wave interactions) (for more details see Supplementary Material). All PROCESS analyses were performed controlling for the parenting styles others the ones entered as the predictor and the moderator and for father and mother perpetrated IPV and child networks use (which resulted to differ across gender).

In order to address non-normality that is common in CDA and IPV data, the bootstrap technique (*N* = 5,000) was used to compute CIs.

## Results

### Preliminary Results

When compared to women, on average men reported that their mother had been more permissive (*M* = 2.58 and 2.41; *t*-test (349) = 2.062, 95% CI [0.01; 0.33]) and their father had perpetrated less IPV (*M* = 1.46 and 1.87; *t*-test (349) = −3.148, 95% CI [−0.64; −0.17]). As concerns CDA, men resulted to perpetrate more aggression (*M* = 1.22 and 1.10; *t*-test (349) = 2.855, 95% CI [0.04; 20]) and less control (*M* = 1.57 and 1.82; *t*-test (349) = −3.034, 95% CI [−0.42; −0.09]) and to suffer more control (*M* = 1.70 and 1.45; *t*-test (349) = 2.924, 95% CI [0.08; 0.43]) and more aggression (*M* = 1.22 and 1.12; *t*-test (349) = 2.611, 95% CI [0.03; 0.18]) than women did (see Supplementary Table 2 for descriptive statistics and correlations).

### Unique Relations of Parenting Styles With Cyber Dating Abuse

Regression models indicated that mother but not father parenting styles were uniquely but weakly related to their child CDA (see Table 1). In particular, the more the mother was perceived as authoritarian the more the child perpetrated and suffered cyber dating control; also, the more the mother was judged as permissive the more the child perpetrated cyber dating aggression and suffered cyber dating control. PROCESS Model 1 revealed that none of the unique associations between parenting styles and CDA was moderated by the gender of participants.

**TABLE 1:** The role of parenting styles on CDA when controlling for networks use and IPV perpetrated by parents.

	Perpetrated CDA–control				Perpetrated CDA–aggression				Suffered CDA–control				Suffered CDA–aggression			
	*R*^2^ = 0.09, *p* = 0.000 Cohen’s *f*^2^ = 0.10				*R*^2^ = 0.07, *p* = 0.005 Cohen’s *f*^2^ = 0.08				*R*^2^ = 0.06. *p* = 0.017 Cohen’s *f*^2^ = 0.06				*R*^2^ = 0.06, *p* = 0.016 Cohen’s *f*^2^ = 0.06			
	B	SE	β	95% CI	B	SE	β	95% CI	B	SE	B	95% CI	B	SE	β	95% CI
M Authoritative PS	–0.01	0.04	–0.02	[−0.08; 0.05]	0.01	0.02	0.04	[−0.02; 0.05]	0.01	0.04	0.03	[−0.07; 0.10]	0.00	0.03	0.01	[−0.05; 0.05]
M Authoritarian PS	0.09	0.05	**0.14**	[0.01; 0.18]	0.02	0.02	0.07	[−0.02; 0.07]	0.11	0.05	**0.17**	[0.01; 0.23]	0.03	0.03	0.09	[−0.02; 0.09]
M Permissive PS	–0.01	0.07	–0.01	[−0.14; 0.13]	0.06	0.03	**0.13**	[0.01; 0.14]	0.16	0.07	**0.15**	[0.01; 0.36]	0.05	0.04	0.11	[−0.01; 0.13]
F Authoritative PS	0.03	0.03	–0.01	[−0.0; 3.09]	0.01	0.02	0.05	[−0.01; 0.04]	–0.02	0.04	–0.06	[−0.10; 0.06]	0.00	0.02	–0.01	[−0.04; 0.04]
F Authoritarian PS	–0.02	0.04	0.06	[−0.10; 0.07]	0.00	0.02	–0.01	[−0.04; 0.04]	–0.03	0.04	–0.05	[−0.14; 0.08]	0.01	0.02	0.04	[−0.04; 0.05]
F Permissive PS	0.09	0.07	–0.03	[−0.05; 0.24]	0.00	0.03	–0.01	[−0.06; 0.06]	–0.03	0.07	–0.03	[−0.17; 0.10]	0.01	0.04	0.02	[−0.06; 0.08]
IPV perpetrated by M	0.09	0.06	0.09	[−0.06; 0.24]	0.08	0.03	**0.18**	[0.02; 0.18]	–0.05	0.06	–0.05	[−0.17; 0.08]	0.04	0.03	0.10	[−0.01; 0.11]
IPV perpetrated by F	0.07	0.05	0.10	[−0.06; 0.21]	0.01	0.02	0.03	[−0.07; 0.10]	0.04	0.05	0.05	[−0.08; 0.17]	0.00	0.03	–0.01	[−0.06; 0.06]
Networks use	0.08	0.03	**0.14**	[0.03; 0.14]	0.01	0.02	0.03	[−0.02; 0.04]	0.05	0.05	0.08	[−0.01; 0.11]	0.00	0.02	0.05	[−0.02; 0.05]

*M, mother; F, father; PS, parenting style; IPV, intimate partner violence; CDA, cyber dating abuse.*

*Significant results are typed in bold.*

### Joint Relations of Parenting Styles With Cyber Dating Abuse

From PROCESS Model 1, we found that only the mother authoritarian style and the father permissive style interacted in relating to their children CDA. Specifically, the association of mother authoritarian style with both perpetrated and suffered cyber dating aggression was stronger the more permissive the father was (2-wave interaction effects: *B* = 0.06, β = 0.13, 95% CI [01, 0.10], *f*^2^ = 0.02^1^ and *B* = 0.05, β = 0.14, 95% CI [0.01, 0.10], *f*^2^ = 0.02 for perpetrated and suffered aggression, respectively). Simple slope tests showed that such associations were significant only for children having a more permissive father (1 SD above the mean) (*B* = 0.06, β = 0.18, 95% CI [0.01, 0.11] and *B* = 0.06, β = 0.21, 95% CI [0.01, 0.11] for perpetrated and suffered aggression, respectively).

**FIGURE 1:**
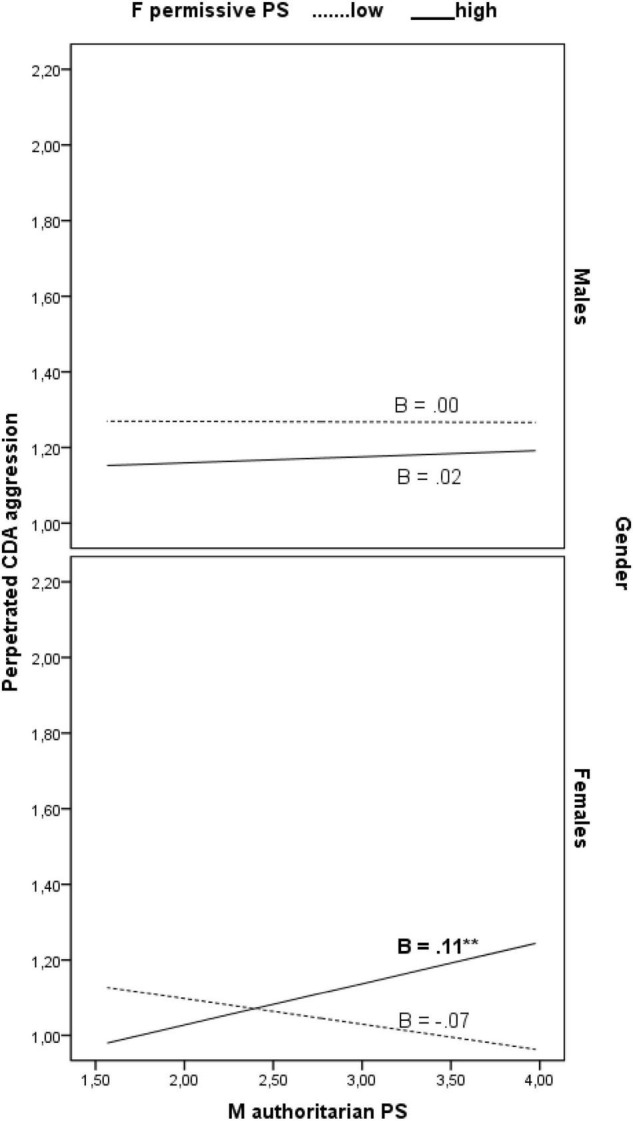
The conditional effects of mother authoritarian parenting style on perpetrated cyber dating aggression as a function of father permissive parenting style and child gender. **p < 0.01. M, mother; F, father; PS, parenting style; CDA, cyber dating abuse.

Finally, PROCESS Model 3 showed that mother and father parenting styles interacted in relating to CDA differently for daughters and sons. Specifically, the previous interaction effects of mother authoritarian style and father permissive style on perpetrated cyber dating aggression were significantly moderated by child gender (3-wave interaction effects: *B* = 0.11, β = 0.13, 95% CI [0.02, 0.19], *f*^2^ = 0.02). Simple slope test showed that mother authoritarian style was significantly associated with a higher degree of perpetrated cyber dating aggression only in daughters having a more permissive father (1 SD above the mean) (*B* = 0.11, β = 0.34, 95% CI [0.04, 0.18]) (see Figure 1).

**FIGURE 2:**
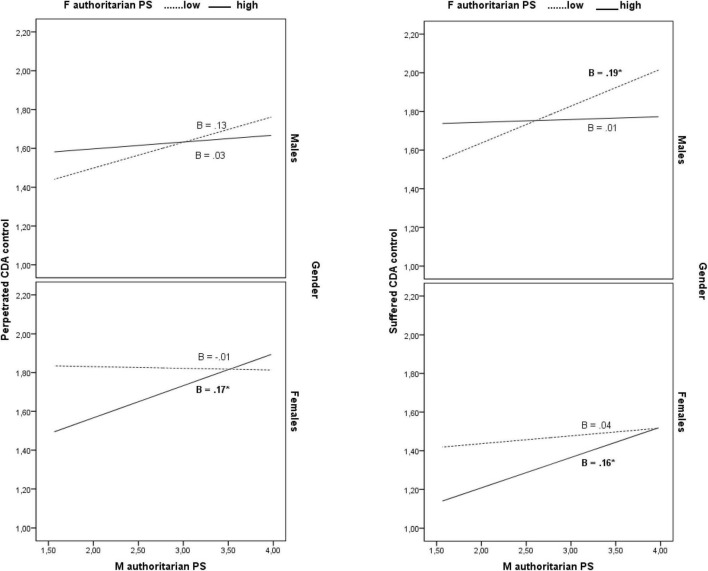
The conditional effects of mother authoritarian parenting style on perpetrated and suffered cyber dating control as a function of father authoritarian parenting style and child gender. *p < .05. M, mother; F, father; PS, parenting style; CDA, cyber dating abuse.

In addition, the association of mother authoritarian style with perpetrated and suffered cyber dating control varied as a function of both father authoritarian style and child gender (3-wave interaction effects: *B* = 0.10, β = 0.10, 95% CI [01, 0.19], *f*^2^ = 0.01 and *B* = 0.10, β = 0.11, 95% CI [01, 0.20], *f*^2^ = 0.01 for perpetrated and suffered control, respectively). Simple slope tests showed that mother authoritarian style was significantly associated with higher degrees of perpetrated and suffered cyber dating control in daughters having more authoritarian fathers (1 SD above the mean) (*B* = 0.17, β = 0.25, 95% CI [0.03, 0.30] and *B* = 0.16, β = 0.10, 95% CI [0.02, 0.30] for perpetrated and suffered control, respectively). In addition, mother authoritarian style was significantly associated with a higher degree of suffered cyber dating control in sons having poorly authoritarian fathers (1 SD below the mean) (*B* = 0.19, β = 0.28, 95% CI [0.03, 0.35]) (see Figure 2).

**FIGURE 3:**
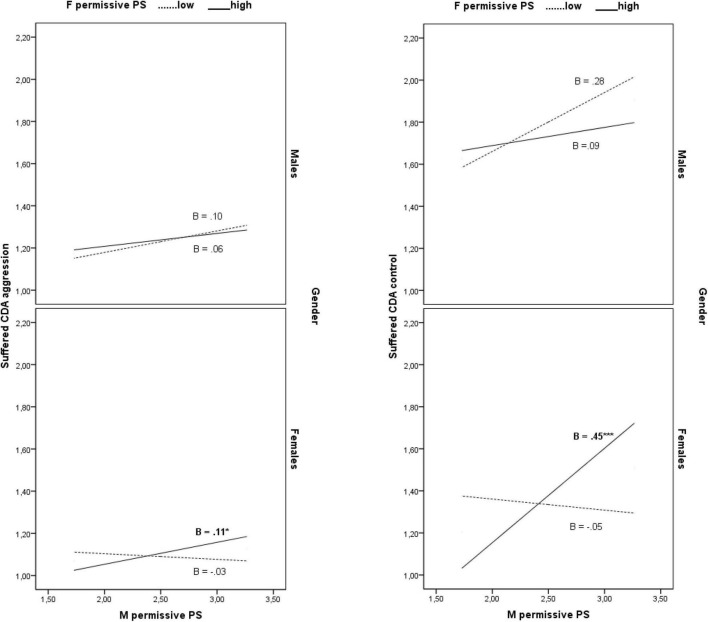
The conditional effects of mother permissive parenting style on suffered cyber dating aggression and control as a function of father permissive parenting style and child gender. *p < .05, ***p < .001. M, mother; F, father; PS, parenting style; CDA, cyber dating abuse.

Finally, mother permissive style was differently related to suffered cyber dating aggression and control as a function of father permissive style and child gender (3-wave interaction effects: *B* = 0.11, β = 0.09, 95% CI [0.01, 0.22], *f*^2^ = 0.01 and *B* = 0.46, β = 0.17, 95% CI [0.22, 0.70], *f*^2^ = 0.04 for suffered aggression and suffered control, respectively). Simple slope tests showed that mother permissive style was significantly associated with higher degrees of suffered cyber dating aggression and control only in daughters having more permissive fathers (1 SD above the mean) (*B* = 0.10, β = 0.22, 95% CI [0.01, 0.21] and *B* = 0.45, β = 0.42, 95% CI [0.22, 0.68] for suffered aggression and suffered control, respectively) (see Figure 3).

## Discussion

Our results showed that the more young adults reported that their mothers had been authoritarian or permissive during their childhood the more likely they were to be involved in a cyber abusive dating relationship. In fact, when controlling for the confounding effects of IPV and networks use, mothers’ authoritarian parenting was uniquely, albeit weakly, associated to their children’s perpetration and victimization of cyber dating control. Partially in line with our prediction (H1), these results support the expected relation between authoritarian parenting and CDA, but only when mothers’ parenting and the control dimension of CDA were considered. Thus, young adults raised by more authoritarian mothers (who were coercive and controlling, but poorly empathic and warm) tend to replicate and bear controlling practices when interacting online with their partners. According to social learning theory (Bandura, 1977), children who are exposed to controlling parents may view their parents’ behaviors as acceptable or desirable and model their interpersonal behaviors based on them, therefore engaging more controlling behaviors with their partner and tolerating more controlling behaviors by him/her (Curry and Zavala, 2020). Alternatively, attachment theory (Bowlby, 1969) posits that coercive family processes facilitate the development of insecure attachment, which in turn contributes to personality characteristics, such as separation anxiety, partner jealousy, and distrust, which likely increase partner surveillance (Guerrero, 1998; Mikulincer and Shaver, 2010; Buck et al., 2012).

Moreover, mothers’ permissive parenting was uniquely, albeit weakly, associated to their children’s perpetration of cyber dating aggression and victimization of cyber dating control. Young adults raised by permissive parents are less used to be controlled and, because of the few guidelines and limited rules received, tend to be more impulsive, lacking self-regulation and self-control (Patock-Peckham et al., 2001; Piotrowski et al., 2013). These features might expose them to a higher risk of acting aggressively toward their partner not only offline (Pinquart, 2017) but also online, and of overestimating and poorly bearing their partner’s control.

Regarding authoritative parenting, contrary to the literature on offline externalizing and abusive behaviors (Pinquart, 2017), but consistent with a growing line of research that questions the protective role of the authoritative style in relation to cyber aggression (Muñiz-Rivas et al., 2019; Zhang et al., 2021), we found that this parenting style was not uniquely related to children’s CDA perpetration and victimization. Possibly, other variables which were not considered in this study, such as parent-child communication about affective relations and risks and opportunities of new technologies, might moderate the relationship between authoritative parenting and CDA.

Overall, mothers’ authoritarian and permissive parenting practices related more strongly to their children’s involvement in cyber abusive relationships than fathers’ parenting practices. This finding supports our hypothesis (H2) and Muñiz-Rivas et al.’s (2019) results and can be explained by the primary role mothers are expected to play in children rearing, especially in the areas of effective relationships. Indeed, consistent with the dominant gendered expectations and the ideology of “intensive mothering” (Hays, 1996), mothers are, willingly or not, still the primary caregiver in the family (Raley et al., 2012; Carlson et al., 2016) and feel to be the main responsible for their children development and outcomes. Fathers generally have less responsibility for their adolescent children’s discipline, daily care, and recreational activities and are also less involved in their children’s peer relations (Updegraff et al., 2001; Phares et al., 2009). This evidence calls for a more egalitarian upbringing.

The unique relations of mothers’ and fathers’ parenting styles with their children perpetrated and suffered CDA were not moderated by children gender, thereby disconfirming our hypothesis (H3) and suggesting that other factors may explain gender differences in CDA, such as hegemonic masculinity and sexual aggression myths (March et al., 2021). This result is consistent with a recent meta-analysis that found no moderating effect of gender on the relationship between parenting styles and children’s offline externalizing problems (Pinquart, 2017).

Even though not uniquely associated to their children’s CDA, fathers’ parenting styles do interact with mothers’ parenting styles in relating to their daughters’ CDA. Specifically, mothers’ authoritarian style positively related to their daughters perpetrated and suffered cyber control only if fathers were authoritarian; similarly, mothers’ permissive style was positively related to their daughters suffered cyber aggression and control only if fathers were permissive. Consistent with previous evidence (McKinney and Renk, 2008), these findings suggest that congruence in parenting is not necessarily related to beneficial outcomes: when fathers and mothers consistently adopt dysfunctional parenting strategies, their daughters, who usually internalize parents’ standards, values, and viewpoints more than sons do (Zentner and Renaud, 2007), might be exposed to a higher risk of perpetrating and suffering CDA.

Moreover, the mothers’ authoritarian style was positively related to their daughters perpetrated cyber aggression and to their sons suffered cyber control only if fathers were, respectively, permissive and poorly authoritarian. Therefore, in line with previous research (Ruiz-Hernández et al., 2019), parental inconsistency in parenting styles seems to have detrimental implications for the involvement of children in cyber abusive relationships, especially when it combines two dysfunctional parenting styles.

When interpreting these results, several limitations of the study and avenues for future research should be considered. First, the small sizes of effects call for larger and more heterogeneous samples to reach more definitive and generalizable conclusion. Second, the cross-sectional design does not provide information on the direction of effects, to explore the which will be important to collect longitudinal data. Third, the children’s retrospective perceptions of parenting practices may be different from those actually implemented, therefore the use of observational measures or multi-informant reports that assess parenting practices when they display should be preferred in the future. Finally, given that the different families to which daughters and sons belong may be a confounder of the gender differences that emerged, data provided by male and female siblings from the same family should be collected to reach a better understanding of these differences.

Notwithstanding these limitations, this study made significant contributions to the literature on the role of gendered-differentiated family socialization in the development of cyber abusive romantic relationships in young adulthood. In particular, it shows that specific maternal and paternal parenting styles have not only unique but also complex joint relations with cyber dating aggression and control perpetrated and suffered by their children and that these relations significantly differ across sons and daughters. These findings have also interesting practical implications for educational programs aimed at improving parenting style (for a review see Ryan et al., 2017). Specifically, they suggest that such programs might be more effective when they not only involve both parents but also intervene on each parent’s style according to the other parent’s style and to the child’s sex. Our results might also help parents to become more aware of the wide-ranging impact of their parenting practices on children’s offline and online behaviors, and more motivated to get involved in parenting interventions when offered to them.

## Data Availability Statement

The raw data supporting the conclusions of this article will be made available on request to the corresponding author.

## Ethics Statement

Ethical review and approval was not required for the study on human participants in accordance with the local legislation and institutional requirements. The patients/participants provided their written informed consent to participate in this study.

## Author Contributions

FP and DG designed the study. MP, LC, and DG collected the data, and FP analyzed them. All co-authors participated in the discussion of the results, drafted the manuscript, and approved it for publication.

## Conflict of Interest

The authors declare that the research was conducted in the absence of any commercial or financial relationships that could be construed as a potential conflict of interest.

## Publisher’s Note

All claims expressed in this article are solely those of the authors and do not necessarily represent those of their affiliated organizations, or those of the publisher, the editors and the reviewers. Any product that may be evaluated in this article, or claim that may be made by its manufacturer, is not guaranteed or endorsed by the publisher.

## Footnotes

1 *f*^2^ effect sizes of 0.02, 0.15, and 0.35 can be considered small, medium, and large, respectively (Cohen, 1988). However, when interpreting the interaction term’s impact, these cutoffs result overly conservative and are commonly replaced by *f*^2^ values of 0.005, 0.01, and 0.025 (Aguinis et al., 2005; Kenny, 2018).
