# Supplementary material for: Gender Differences in the Associations Between Perceived Parenting Styles and Young Adults’ Cyber Dating Abuse
Source: Front Psychol. 2022 Mar 24;13:818607. doi: 10.3389/fpsyg.2022.818607 (PMC8987230; doi:10.3389/fpsyg.2022.818607)
Supplement: Supplementary file 1 [file Table_1.pdf]

Supplementary materials

Data analyses details

Multiple regression models were run to estimate the extent to which parenting styles were uniquely associated with each CDA dimension when controlling for the confounding effects of IPV and networks use (which resulted to differ across gender). PROCESS Models 1 with participants' gender of participants as moderator were employed to test whether the above unique associations differed across gender. PROCESS Models 1 in which each father parenting style was entered as a moderator of the association that each mother parenting style had with each dimension of child CDA were used to verify whether father and mother parenting styles were jointly associated with child CDA. PROCESS Models 3 with gender of participants as additional moderator were employed to test whether the above joint associations differed across gender.

Table 1. Cronbach Alpha Coefficient Values for the Study Variables

Variable	Cronbach Alpha		
	Overall sample	Males	Females
M Authoritative PS	.98	.97	.98
M Authoritarian PS	.92	.91	.92
M Permissive PS	.80	.79	.80
F Authoritative PS	.98	.98	.98
F Authoritarian PS	.94	.94	.94
F Permissive PS	.77	.78	.77
IPV perpetrated by M	.71	.70	.72
IPV perpetrated by F	.84	.83	.84
Perpetrated CDA - control	.86	.87	.86
Perpetrated CDA - aggression	.84	.81	.89
Suffered CDA - control	.89	.89	.89
Suffered CDA - aggression	.78	.74	.83

Note. M = Mother; F = Father; PS = Parenting Style; IPV = Intimate Partner Violence; CDA = Cyber Dating Abuse.

Table 2. Descriptives and Correlations among the Study Variables for Males (above the diagonal) and Females (below the diagonal)

	1.	2.	3.	4.	5.	6.	7.	8.	9.	10.	11.	12.	M	SD
1. M Authoritative PS	-	-.37**	-.18*	.68**	-.14	-.16*	-.35**	-.23**	-.09	-.08	-.07	-.10	4.47	1.44
2. M Authoritarian PS	-.60**	-	.20**	-.24**	.48**	.23**	.40**	.37**	.25*	.10	.16*	.18*	2.78	1.16
3. M Permissive PS	-.13	.15	-	-.20**	.23**	.69**	.11	-.04	.11	.09	.10	.18*	2.58	.75
4. F Authoritative PS	.48**	-.20**	-.17*	-	-.27**	-.11	-.21**	-.30**	-.09	-.01	-.12	-.10	3.83	1.54
5. F Authoritarian PS	-.22**	.35**	.14	-.47**	-	.14	.27**	.47**	.13	.07	.07	.09	2.66	1.31
6. F Permissive PS	-.04	.16*	.41**	.03	-.08	-	.11	-.04	.13	.01	.06	.14	2.47	.74
7. IPV perpetrated by M	-.19*	.30**	.15*	.10	.16*	.32**	-	.56**	.19**	.20**	.04	.20**	1.49	.84
8. IPV perpetrated by F	-.18*	.15*	.13	-.45	.52**	.04	.38**	-	.20**	.03	.18	.12	1.46	.94
9. Perpetrated CDA - control	-.12	.18*	.10	.02	.05	.19*	.20*	.04	-	.40**	.55**	.28**	1.57	.72
10. Perpetrated CDA - aggression	.01	.16	.18*	.00	.09	.21**	24**	.22**	.57**	-	.19*	.41**	1.22	.40
11. Suffered CDA - control	-.13*	.18*	.21**	-.07	.11	.09	.03	.00	.28**	.33**	-	.55**	1.70	.89
12. Suffered CDA - aggression	-.09	.14	.12	-.05	.16*	.08	.11	.10	.25**	.67**	.57**	-	1.22	.38
M	4.48	2.76	2.41	3.80	2.75	2.37	1.52	1.87	1.82	1.10	1.45	1.12		
SD	1.58	1.26	.77	1.80	1.51	.77	,83	1.27	,84	.36	.74	.33		

Note. **p<.01, *p<.05. M = Mother; F = Father; PS = Parenting Style; IPV = Intimate Partner Violence; CDA = Cyber Dating Abuse. Possible range of response: 1 – 7 for all scales
